# Validation of the Evidence-Based Practice Competence Questionnaire for Nursing Students: A Cross-Sectional Study in Greece

**DOI:** 10.3390/nursrep11040073

**Published:** 2021-10-03

**Authors:** Athina Patelarou, Stefania Schetaki, Konstantinos Giakoumidakis, Paschalina Lialiou, Evridiki Patelarou

**Affiliations:** 1 Department of Nursing, Faculty of Health Sciences, Hellenic Mediterranean University, Heraklion, 71004 Crete, Greece; stef.schetaki@gmail.com (S.S.); epatelarou@hmu.gr (E.P.); 2 NIMTS Veterans’ Fund Military Hospital, 11521 Athens, Greece; kongiakoumidakis@gmail.com; 3 Korgialenio-Benakio Hospital, 11526 Athens, Greece; plialiou@nurs.uoa.gr

**Keywords:** evidence-based practice, nursing, students, validity

## Abstract

(1) Background: evidence-based nursing has been widely adopted by healthcare facilitators, and it is predicated on the connection between research evidence and clinical practice. The knowledge and implementation of evidence-based practice (EBP) depend on a variety of long-established barriers and attitudes. The purpose of this study was to translate and validate the Greek version of the evidence-based practice competence questionnaire (EBP-COQ) and test it on a sample of Greek undergraduate nursing students. (2) Methods: a cross-sectional analysis was conducted on data obtained between November 2018 and January 2019. A convenience sample of 320 Greek undergraduate nursing students participated in a survey to examine the psychometric properties of the tool. The reliability and validity of the tool was examined. Cronbach’s coefficient alpha was used to determine the scale’s internal consistency reliability. (3) Results: the EBP-COQ was translated and validated. The estimated Cronbach’s alpha was higher than 0.70 for all scales. Of the participants, 22.5% were men and 77.5% were women, while 31% of them were in their final year of nursing education. A total of 61% of the students stated that they had not participated in an EBP seminar in the past. High Spearman’s correlation coefficients were found for “Knowledge in EBP” with “Personal attitude towards EBP” (rs = 0.329, *p* < 0.001). (4) Conclusions: the Greek version of the EBP-COQ is a valid instrument that can be used in the Greek population. It provides information about attitude, knowledge and skills in the EBP approach.

## 1. Introduction

Evidence-based practice (EBP) is an approach that guides the decision-making of healthcare personnel to use the best available research evidence along with clinical expertise, and the unique values and preferences of the patient [1]. The main health professional organizations have set EBP as an important element of quality care, establishing EBP competency as a professional standard [2,3,4,5,6]. However, even though nurses may have a positive attitude towards EBP, they have a lack of knowledge and skills [7], and do not feel equipped enough to implement EBP. Despite the educational background of nurses being the cornerstone of EBP promotion, a vast amount of research studies have focused on the effectiveness of EBP teaching strategies for undergraduate health students [8]. Furthermore, the need for sufficient EBP integration into undergraduate nursing programs has been strongly suggested [9,10]. Nursing students, as future nurses providing high-quality care, need to be well prepared and upskilled to embrace EBP into clinical practice, therefore they should be immersed in the field of EBP early in their undergraduate studies. The EBP competence of nursing students has been investigated in many recent studies, revealing a low to moderate EBP level of competence and a variation in the attitudes of nursing students towards EBP [11,12].

In Greece, there is a lack of research tools to investigate nursing students’ EBP competence, and this is the main obstacle for research in Greek educational institutions [7]. The literature supports the view that the evidence-based practice competence questionnaire (EBP-COQ) [12] is a valid scale with adequate psychometric properties, and it has been used as research tool in different contexts in Spain [13], Latin America [14], India, Saudi Arabia, Nigeria and Oman [14,15], and has been translated and validated in many different languages, such as Turkish [16], Italian [17], Polish [18] and Chinese [19]. A recent scoping review concluded that the main barriers to the implementation of EBP by health professionals are workload, an unsupportive professional environment and culture, a lack of resources and a lack of authority to change established practices [20]. To the best of our knowledge, there is a lack of research exploring the readiness of nursing staff to implement EBP practices in Greece. The main purpose of this study was to fill this existing gap by introducing an instrument to assess EBP that is already used by other nurses worldwide. The primary purpose of this study was to translate and validate the Greek version of the EBP-COQ and to test its psychometric properties and factor structure on a sample of Greek undergraduate nursing students.

## 2. Materials and Methods

### 2.1. Design and Setting

A cross-sectional, psychometric, validation study was conducted at the Nursing Department of the Hellenic Mediterranean University in Greece.

### 2.2. Data Collection and Sample of the Study

Data collection took place from November 2018 to January 2019. A convenience sample of undergraduate nursing students was used, the characteristics of which are presented below (Table 1).

### 2.3. Data Collection Instruments

A questionnaire was used as the instrument of the study. The first part of the questionnaire included questions on demographics, gathering information on things such as age, gender and educational level. The second part included the EBP-COQ. The EBP-COQ was created, developed and validated in the Spanish language [11]. It explores personal attitudes toward EBP knowledge and skills, English language, previous education in EBP or in research methods, statistics and computer skills.

Furthermore, it consists of 25 items in which respondents specify their level of agreement to a statement in a five-point Likert scale (1 to 5): 1 = totally disagree; 2 = disagree; 3 = neither agree nor disagree; 4 = agree; and 5 = totally agree. Eight of the items have reverse scoring and a principal component analysis, resulting in three-dimensional components. The first dimension consists of 13 items labeled “Attitudes toward EBP”, and the second and third dimensions are “Skills in EBP” (6 items) and “Knowledge in EBP” (6 items), respectively. Additionally, there is a visual analogue scale (VAS) to explore participants’ self-reported perceptions of the EBP-COQ (Personal attitude towards EBP, Skills in EBP, Knowledge in EBP, Attitude towards EBP promotion and Colleagues’ attitudes towards EBP).

### 2.4. Research Ethics

Permission to use the questionnaire was obtained from the original author of the instrument. This research was approved by the Hellenic Mediterranean University Ethics Committee. Respondents were informed via an information sheet on the purpose of the research, asking them to give their full participation consent. The research respected the dignity of the participants, protected their privacy and anonymity, and ensured an adequate level of confidentiality. The data were used only for the purposes of the present study.

### 2.5. The Linguistic Validity of the Scale

The Spanish version of the EBP-COQ has demonstrated very good psychometric properties, such as reliability and validity [11]. The estimated Cronbach alpha values of the 25 items in the Spanish version were ranked at 0.888, while the subscales shown in Table 2 were ranked at 0.940, 0.756 and 0.800.

The original version of the EBP-COQ was translated into the Greek language using the back-translation strategy for cross-cultural research, in line with the World Health Organization protocol “Process of translation and adaptation of instruments” [20]. First, two experienced bilingual translators independently performed a forward translation from the original Spanish version. One translator was an advanced practice nurse and the other was a physician. Both forward-translated versions were then reconciled and incorporated into the Greek version by an expert panel, using a consensus procedure. The expert panel consisted of four clinical nurses, three professors of nursing, a psychologist and an expert in psychometrics. The expert panel checked all items and inserted their recommendations into the questionnaire. The back translation was carried out by a Spanish teacher who was qualified in the Greek language, but had no knowledge of the EBP-COQ or access to the original Spanish version. The semi-final version was derived from a reconciliation of the original back and forward translations. Finally, the Greek version of the instrument was considered to be correct as it was in agreement with the original Spanish version.

### 2.6. Data Analysis

The psychometric properties of the EBP-COQ were investigated. The mean score and standard deviation were used to express the measured EBP-COQ scales, while frequency and % frequency were used for categorical and discrete numerical variables. Missing values, extreme measures (floor/ceiling effects), internal consistency (Cronbach’s alpha) and factor analysis (principal component analysis (PCA)) were some of the psychometrics that were measured. Regarding the PCA, the Kaiser–Meier–Olkin measure was applied for measuring model fitting. A varimax rotation was included and the results of the model validation were based on the reported eigen values and % explained variance.

The external validity was measured with Pearson’s or Spearman’s coefficients according to the data normality. The discriminant ability was tested using the independent samples t-test and the non-parametric Mann–Whitney test. Exploration of the differences in a continuous variable in more than two groups was assessed with a one-way ANOVA or the corresponding non-parametric Kruskal–Wallis test. The Kolmogorov–Smirnov test for normality was applied, and all analysis was performed using the IBM SPSS Statistics 24.0 statistical package (IBM Corp., Armonk, NY, USA).

## 3. Results

### 3.1. Sampling

In Table 1, the demographic characteristics (sex, age and academic year) are presented. From an initial set of 356 students, a total of 36 participants (10.1%) were excluded, mainly for incorrect filling or non-valid answers. The analyzed sample (320 students) were mainly women (*n* = 248, 77.5%), while most of the students were younger than 22 years of age (21–22: *n* = 137, 42.8%, 18–20: *n* = 121, 37.8%). A total of 100 students (31.2%) were in their fourth, and final, academic year of study, while only 31 students (9.7%) were in their third academic year.

In addition, Table 1 presents the respondents’ replies in relation to their educational background in research methods as well as in EBP. Of the respondents, 6.7% (*n* = 20) reported that they held an additional degree, while 175 students (61.0%) stated that they had not had any exposure to EBP. A total of 68.9% (*n* = 202 students) replied that they had not had any experience using research methods, while 80.5% (*n* = 247) had not read more than two research articles. The scores from the VAS scales that explore the self-reported perceptions in the EBP-COQ (Personal attitude towards EBP, Skills in EBP, Knowledge in EBP, Attitude towards EBP promotion and Colleagues’ attitudes towards EBP) are depicted in Figure S1a as box and whisker plots. The highest median value was observed for Personal attitude towards EBP (median = 7.0), while lower medians were observed for Colleagues’ attitudes towards EBP and Skills in EBP (median = 5.0).

The VAS scales related to knowledge of the English language, computer and statistics skills are presented in Figure S1b. The highest median value in the VAS scales was for knowledge of the English language (median = 8.0), followed by knowledge in computers (median = 7.0) and statistics skills (median = 5.0). Table 2 shows some of the results for EBP-COQ items A9, A10 and A11, and more than 15% of participants stated that they “totally disagree” with these items (A9:26.6%, A10:16.2% and A11:22.4%). In addition, more than 30% of participants stated that they “totally agree” with items A1, A6 and A7 (A1: 30.1%, A6: 33.7% and A7: 37.6%).

### 3.2. Construct Validity

A principal component analysis was applied to explore the structural validity of the EBP-COQ Greek version (EBP-COQ_GR). The KMO and Bartlett’s test results were 0.803 (KMO) and χ^2^ = 2450, df = 300, *p* < 0.001 (Bartlett). In the component matrix, all values were higher than 0.500. The eigen values after varimax rotation were much greater than 1.5 (3.211 the lower), and the total cumulative variance explained was 44.837%.

The loadings of each item on each component are summarized in Table 3. Differences were observed in comparison with the Spanish tool as the loading of each item on the resulting PCA components showed a different pattern to that of the developers. In detail, the developer’s EBP scale items A2 and A9–A11 were not loaded on Component 1. Furthermore, item A2 (loading 0.54) was grouped in the new Component 2 as it derived from the PCA, and items A9–A11 were loaded on the new Component 3. Items on the developer’s EBP scales were loaded on the new derived Components 2 and 3. A pattern was observed that the “positive” expressed items were in Component 2 and the “negative” expressed items were in Component 3 (items A9–A11, H2–H3, H5, C3, C5).

The independence of the three components was found to be supported from the correlation coefficients between the 1st, 2nd and 3rd factor (Table S1). No significant correlations were observed between scales 2 and 3 (Pearson’s r = −0.015, *p* = 0.786, Spearman’s r = −0.058, *p* = 0.304). Other variable pairs showed a significant (*p* < 0.05) correlation but the absolute rs did not exceed rs = 0.310.

### 3.3. Internal Consistency: Cronbach’s Alpha Values

The analysis resulted into three factors (Attitude towards EBP, Knowledge-Skills in EBP and EBP Perceptions). Estimated Cronbach’s alpha (Table 4) showed values >0.700 for all scales and total number of items. More specifically, Cronbach’s alpha was 0.811 for all items, 0.858 for Attitude towards EBP, 0.789 for Knowledge-Skills in EBP and 0.777 for EBP Perceptions.

### 3.4. External Validity

The latest produced scales of the Greek version of the EBP-COQ were tested for their differences with related EBP questions. All EBP-COQ_GR scales showed significant correlation with scales related to perceptions of EBP, with the only exceptions being EBP Perceptions and Colleagues’ attitudes towards EBP (rs = −0.048, *p* = 0.395).

High Spearman’s correlation coefficients were found for Knowledge in EBP with Personal attitude towards EBP (rs = 0.329, *p* < 0.001) and with Attitude towards EBP promotion (rs = 0.326, *p* < 0.001). Knowledge-Skills in EBP showed a higher correlation coefficient (rs) with Skills in EBP (rs = 0.465, *p* < 0.001) and Knowledge in EBP (rs = 0.446, *p* < 0.001). Weak but significant correlation coefficients (rs < 0.200, *p* < 0.001) were found in EBP Perceptions and Personal attitude towards EBP.

The results in Table S2 highlight that the VAS scales for other knowledge scales (English language, computer skills and statistics) showed weak correlations. More specifically, EBP Perceptions showed a weak correlation (rs = 0.225, *p* < 0.001) with knowledge in English language and computer skills (rs = 0.160, *p* = 0.004).

### 3.5. Discriminant Ability

The differences in the Greek version of the EBP-COQ scales between parameters of knowledge are presented in Table S3. All scales showed significant differences between sex, existence of EBP practice and knowledge of research methods, apart from sex effect in EBP attitudes (*p* = 0.164).

Men showed higher mean values in Knowledge-Skills in EBP (28.2 ± 4.3) in comparison to women (26.1 ± 5.3) (*p* = 0.003). In contrast, the mean values in EBP Perceptions were higher in women (25.7 ± 5.3) than in men (22.9 ± 6.2) (*p* < 0.001).

Students who had received education in EBP showed higher mean values (36.0 ± 5.3, 27.5 ± 5.0 and 26.3 ± 5.8) in Attitude towards EBP, Knowledge-Skills in EBP and EBP Perceptions (*p* < 0.05).

Students who had been educated in research methods showed higher mean values for each of the EBP scales. More specifically, the mean values for Attitude towards EBP were 35.9 ± 5.6 (with practice) and 34.6 ± 5.7 (without) (*p* = 0.024), while Knowledge-Skills in EBP was 27.6 ± 4.7 (with practice) and 26.0 ± 5.3 (without) (*p* = 0.009). Finally, EBP Perceptions showed higher mean values in students with previous education in research methods (25.9 ± 5.6) in addition to students without such an education (24.6 ± 5.6) (*p* = 0.025).

## 4. Discussion

The EBP-COQ Greek version (EBP-COQ_GR) is a psychometric tool composed of 25-items that is user-friendly and rapidly administered. It explores three main dimensions of EBP expectances: attitudes, skills and knowledge. The main purpose of the present study was to test the validity and reliability of the Greek version of the EBP-COQ. This study was conducted in line with the original study of Ruzafa-Martinez et al. (2013) [11], and was grounded in the quality assurance of the translation and validated in a Greek undergraduate student sample.

The EBP-COQ_GR was tested on internal consistency, construct validity and external validity between independent variables. Standardized Cronbach’s alpha coefficients for the Greek version of the EBP-COQ for all items was up to 0.70 for the three measured factors (Attitude towards EBP, Knowledge-Skills in EBP and EBP Perceptions), meaning that the instrument appears to have high internal consistency, similar to the instrument in the Ruzafa-Martinez et al. study (2013) [11]. In addition, the results of skills and knowledge in a similar study in the Turkish EBP-COQ scale shows lower Cronbach’s alpha values, meaning that skills and knowledge in the EBP of Turkish students can be interpreted as being low [15,16]. However, the Cronbach’s alpha for the entire Turkish questionnaire was found to be 0.826, making it an internally consistent instrument [16].

Results from the descriptive statistics show that there was high correlation between Knowledge in EBP and Skills in EBP. Personal attitude towards EBP is affected by EBP Perceptions. These findings are confirmed by recent studies which documented a positive correlation between EBP attitude and EBP knowledge [16,17,18,19]. In addition, EBP attitude had low correlation with workplace culture and information needs. Moreover, in the present study, speaking fluent English and having computer skills seemed to have an effect on EBP perceptions. Students who were educated in EBP and research methods seemed to have more skills in evidence-based nursing, meaning that education plays a critical role in the implementation of evidence-based practice.

According to the construct validity tests, there were some differences between the two versions. Moreover, in EBP-COQ_GR, Attitude towards EBP is interpreted by items A1, A3–A8, A12 and A13 (factor loadings 0.5–0.75); Knowledge in EBP is interpreted by items A2, H1, H4, H6–C2, C4 and C6 (factor loadings 0.54–0.67); and EBP Perceptions from items A9–A11, H2, H3, H5, C3 and C5 (factor loadings 0.5–0.75). In addition, the developer’s scale items A2 and A9–A11 were not loaded in factor 2 (Skills in EBP), and A9–A11 were loaded in factor 3 (Knowledge in EBP). It is worth noting that the construct of the EBP-COQ_GR is different in comparison to other versions of the tool in other languages. More specifically, the factor loadings and the factors themselves are different, depending on the national culture of each study population [12,13,14,15,16,17,18,19,20].

Furthermore, external contract validity was calculated by exploring the correlation coefficients of the EBP-COQ_GR. High Spearman’s correlation coefficients were found for Knowledge in EBP with Personal attitude towards EBP, and with Attitude towards EBP promotion. This differs from the Spanish version [11], in which a positive and high correlation relationship was found between “Attitude toward research”, “EBP Competence” and “Attitude towards EBP”. However, there was no relationship between “Knowledge in EBP” and “Skills in EBP”; in the Spanish version [11], in addition to the Greek version, those who had been educated in EBP showed a positive attitude, knowledge, skills and perceptions toward EBP. Moreover, students who had educated in research methods showed positive perceptions towards EBP. In comparison with other scale versions [11,12,13], the Polish version presented higher correlation coefficients than the original one [17]. This statement confirms that the EBP-COQ scale is an adaptive scale that can be used in many different languages and samples, producing reliable results related to the evidence-based practice of nurses and students.

This study had certain limitations. First, participants in this study were recruited from only one university from the existing universities in the country, which could impede generalization to other nursing students in other geographical areas. Therefore, further testing of the tool is needed with nursing students from various geographical areas to enhance the reliability. Second, we did not use other research tools in order to estimate convergent and discriminant validity. Finally, we did not use the test-retest reliability method to examine the consistency over time.

To date, there has been limited literature examining nursing personnel’s readiness for, and attitudes toward, evidence-based practice in Greek healthcare settings [6]. Moreover, only a few nursing institutions in Europe have integrated EBP into their nursing curricula [21,22]. This means there is an urgent need for global collaboration in order to develop more EBP courses for nurses and students. The validation of the EBP-COQ_GR instrument comprises the cornerstone of EBP research in nursing staff and promises to fill the gap between knowledge and practice [23]. The dissemination and achievement of evidence-based principles are promising aspects that improve the quality of care and ensure patient and staff safety [24,25].

## 5. Conclusions

In order to assess the readiness of nursing staff for EBP, the present study translated and validated a useful psychometric tool, the Spanish EBP-COQ. The Greek version is a high-quality instrument that is comparable with the original version. The instrument provides information about attitude, knowledge and skills in the EBP domain. It was used on a student sample and constitutes the benchmark for EBP in nursing staff. Future research is needed to determine how nurses approach evidence-based nursing in healthcare practices. The assessment of nursing readiness for EBP will lead the way to filling the educational and administrative gaps that have perpetuated in the nursing profession for years. Finally, this study constitutes the basis for future research in the evidence-based nursing discipline.

## Figures and Tables

**Table 1:** Sample demographics of participants and educational background of the respondents.

		*n*	*n*%
Sex	Man	72	22.5
	Woman	248	77.5
Age categories	18–20	121	37.8
	21–22	137	42.8
	23–24	35	10.9
	25–26	8	2.5
	>26	15	4.7
Academic year	1st	94	29.4
	2nd	95	29.7
	3rd	31	9.7
	4th+	100	31.2
Other degree	No	280	93.3
	Yes	20	6.7
Seminar in EBP (hours)	None	175	61.0
	<40	96	33.4
	40–150	9	3.1
	>150	7	2.4
Research methods (hours)	None	202	68.9
	<40	80	27.3
	40–150	10	3.4
	>150	1	0.3
Research articles (number)	0–2	247	80.5
	3–4	40	13.0
	5–6	7	2.3
	>6	13	4.2

**Table 2:** Summary of responses for the Greek version of the evidence-based practice competence questionnaire (EBP-COQ).

	Totally Disagree		Disagree		Neither Agree nor Disagree		Agree		Totally Agree	
	*n*	%	*n*	%	*n*	%	*n*	%	*n*	%
A1	2	0.6%	9	2.5%	71	19.9%	167	46.9%	107	30.1%
A2	5	1.4%	26	7.3%	129	36.3%	159	44.8%	36	10.1%
A3	4	1.1%	19	5.4%	82	23.4%	172	49.0%	74	21.1%
A4	6	1.7%	27	7.6%	93	26.2%	158	44.5%	71	20.0%
A5	10	2.8%	23	6.5%	81	22.8%	149	41.9%	93	26.1%
A6	5	1.4%	12	3.4%	69	19.5%	148	41.9%	119	33.7%
A7	2	0.6%	9	2.5%	64	18.1%	146	41.2%	133	37.6%
A8	3	0.9%	23	6.7%	81	23.5%	153	44.5%	84	24.4%
A9	93	26.6%	89	25.4%	98	28.0%	51	14.6%	19	5.4%
A10	57	16.2%	106	30.2%	117	33.3%	55	15.7%	16	4.6%
A11	79	22.4%	99	28.1%	106	30.1%	54	15.3%	14	4.0%
A12	3	0.9%	32	9.1%	73	20.7%	148	42.0%	96	27.3%
A13	5	1.4%	20	5.6%	76	21.5%	149	42.1%	104	29.4%
H1	6	1.7%	41	11.6%	123	34.7%	130	36.7%	54	15.3%
H2	19	5.4%	85	24.1%	131	37.1%	86	24.4%	32	9.1%
H3	21	5.9%	103	29.2%	127	36.0%	73	20.7%	29	8.2%
H4	4	1.1%	45	12.9%	129	36.9%	121	34.6%	51	14.6%
H5	25	7.1%	91	25.9%	122	34.7%	92	26.1%	22	6.3%
H6	5	1.4%	42	12.0%	145	41.4%	122	34.9%	36	10.3%
C1	23	6.5%	51	14.4%	119	33.6%	105	29.7%	56	15.8%
C2	34	9.6%	59	16.7%	122	34.6%	99	28.0%	39	11.0%
C3	23	6.5%	100	28.1%	129	36.2%	60	16.9%	44	12.4%
C4	16	4.5%	57	16.2%	136	38.6%	110	31.3%	33	9.4%
C5	23	6.5%	63	17.7%	136	38.2%	102	28.7%	32	9.0%
C6	19	5.4%	61	17.2%	124	34.9%	108	30.4%	43	12.1%

**Table 3:** Factor loading.

		Factor		
		1	2	3
A1	The EBP helps with making decisions in clinical practice.	0.68	0.11	0.05
A2	I am confident that I will be able to critically evaluate the quality of a scientific article.	0.14	0.54	0.03
A3	The practice of EBP will help us have a better definition for the role of nurses.	0.69	0.13	−0.01
A4	Nursing contracts should include time to read scientific papers and make a critical appraisal of them.	0.57	0.08	−0.04
A5	Widespread EBP implementation will allow an increased nursing autonomy from other professions.	0.70	0.09	0.09
A6	When I work as a nurse, I am pleased if an EBP is in practice.	0.69	0.02	−0.07
A7	The application of EBP improves patients’ healthcare outcomes.	0.74	0.01	0.04
A8	In the future, I wish to contribute to applying the EBP.	0.68	0.14	−0.11
A9	I do not like reading scientific articles.	0.34	−0.18	0.57
A10	Patient care will undergo minor changes with the application of EBP.	0.33	−0.30	0.58
A11	It pleases me that the EBP is only a theoretical movement that does not take place in practice.	0.34	−0.32	0.56
A12	If I have the opportunity, I will undertake an EBP course.	0.65	0.03	0.07
A13	I would like to have better access to published nursing scientific evidence.	0.62	0.17	0.05
H1	I feel able to ask a clinical question to start searching for the best scientific evidence.	0.00	0.64	−0.15
H2	I do not feel able to search for scientific evidence in the principal health sciences databases.	0.04	0.10	0.67
H3	I do not feel able to search for scientific information about a subject in the most important bibliographic indexes.	−0.04	0.07	0.75
H4	I feel able to critically evaluate the quality of a scientific article.	0.03	0.62	0.11
H5	I do not feel able to analyze whether the obtained results of a scientific study are valid.	−0.11	0.09	0.62
H6	I feel able to analyze the practical utility of a scientific study.	0.14	0.67	0.08
C1	I know how to make clinical questions organized in the PICO format.	0.18	0.59	0.18
C2	I know the principal sources that offer the revised and catalogued information behind the evidence.	0.16	0.64	0.06
C3	I do not know the most important characteristics of the principal investigation designs.	−0.11	0.09	0.61
C4	I know the different evidence levels of the designs of the investigation studies.	−0.03	0.62	−0.06
C5	I do not know the different recommendation grades regarding the adoption of a determined procedure or health intervention.	−0.12	0.15	0.59
C6	I know the principal measures of association and potential impact that allow us to evaluate the magnitude of the analyzed effect in investigation studies.	0.09	0.64	−0.02

**Table 4:** Cronbach’s alpha coefficients for the EBP-COQ Greek version (EBP-COQ_GR) subscales.

	EBP-COQ_GR	Cronbach’s Alpha	Number of Questions
1	Attitude towards EBP	0.858	9
2	Knowledge-Skills in EBP	0.789	8
3	EBP Perceptions	0.777	8
	Total	0.811	25

## Data Availability

The data presented in this study are available on request from the corresponding author.

## Supplementary Materials

The following are available online at https://www.mdpi.com/article/10.3390/nursrep11040073/s1. Figure S1: (a) Box and Whisker Plots for skills, knowledge and attitude VAS scales, (b) Box and Whisker Plots of VAS scale scores for personal skills on language, computer skills and statistics; Table S1: Pearson’s and Spearmans’ rho coefficients for EBP-COQ_GR questionnaire; Table S2: Correlation of EBP-COQ_GR subscales vs. knowledge in special subjects. Discriminant ability; Table S3: Discriminant ability EBP-COQ_GR vs. sex and education in EBP.
