# Supplementary material for: Validation of the Evidence-Based Practice Competence Questionnaire for Nursing Students: A Cross-Sectional Study in Greece
Source: Nurs Rep. 2021 Oct 3;11(4):765–74. doi: 10.3390/nursrep11040073 (PMC8715452; doi:10.3390/nursrep11040073)
Supplement: Supplementary file 1 [file nursrep-11-00073-s001.zip › nursrep-1331708-supplementary.pdf]

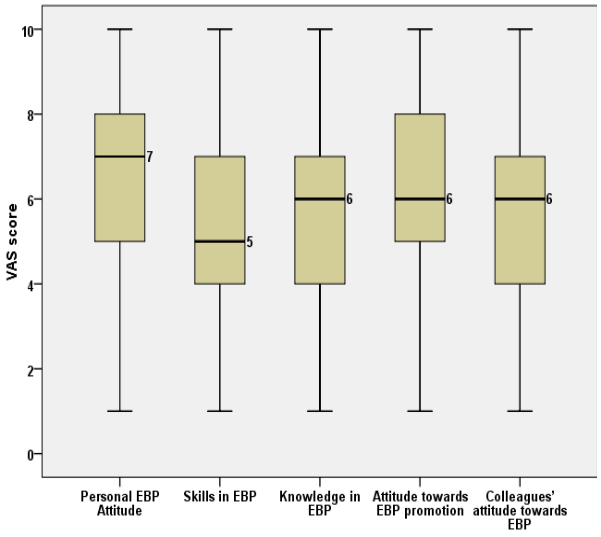

(**a**)

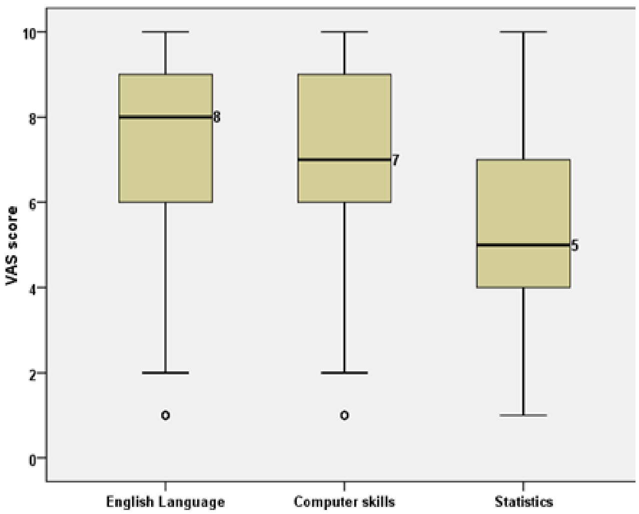

(**b**)

Figure S1. (a) Box and Whisker plots for skills, knowledge, attitude VAS scales; **(b)** Box and Whisker plots of VAS scales scores for personal skills on language, computer skills and statistics

Table S1. Pearson's and Spearmans' rho coefficients for EBP-COQ_GR questionnaire*

Factor		Attitude toward EBP	Knowledge - Skills in EBP	EBP Perceptions
Attitude toward EBP	r	1.000	0.309	0.209
	p	-	<0.001	<0.001
Knowledge - Skills in EBP	r	0.261	1.000	-0.015
	p	<0.001	-	0.786
EBP Perceptions	r	0.192	-0.058	1.000
	p	<0.001	0.304	-

* Up diagonal Pearson's rho coefficients, Down diagonal Spearman's rho coefficients

Table S2. Correlation of EBP-COQ_GR subscales vs knowledge in special subjects. Discriminant ability

Knowledge in		EBP Attitude	Knowledge -Skills in EBP	EBP Perceptions
English language	rs	0.101	0.019	0.225
	p	0.071	0.742	<0.001
Computer Skills	rs	0.108	0.135	0.160
	p	0.054	0.016	0.004

Statistics Skills	rs	0.048	0.262	0.029
	p	0.397	<0.001	0.611

Table S3. Discriminant ability EBP-COQ_GR vs sex, education in EBP

	Attitude toward EBP		Knowledge-Skills in EBP		EBP Perceptions	
**Sex**	**Mean**	**SD**	**Mean**	**SD**	**Mean**	**SD**
Man	34.3	6.2	28.2	4,3	22,9	6,2
Woman	35.3	5.5	26.1	5,3	25,7	5,3
P	0.164		0.003		<0.001	
Education in EBP						
Without	34.3	5.9	25.9	5,2	24,0	5,3
With	36.0	5.3	27.5	5,0	26,3	5,8
P	0.011		0.016		<0.001	
Education in Research Methods						
Without	34.6	5.7	26.0	5,3	24,6	5,6
With	35.9	5.6	27.6	4,7	25,9	5,6
P	0.024		0.009		0.025	
